# SMBE Satellite Meeting on Reticulated Microbial Evolution 2014—Meeting Report

**DOI:** 10.1093/gbe/evu173

**Published:** 2014-08-12

**Authors:** Tal Dagan, Eric Bapteste, James O. McInerney, William F. Martin

**Affiliations:** ^1^Institute of Microbiology, Christian-Albrechts-University of Kiel, Germany; ^2^UMR CNRS 7138 Systématique, Adaptation, Evolution, Université Pierre et Marie Curie, Paris, France; ^3^Department of Biology, National University of Ireland Maynooth, Ireland; ^4^Institute of Molecular Evolution, Heinrich-Heine University Düsseldorf, Germany

The concept of gene transfer among prokaryotes has been around for quite a while. Griffith (1928) transformed an attenuated and nonencapsulated *Pneumococcus* culture (type R) into a fully encapsulated and virulent Pneumococcus strains (type S) long before anyone knew that the material underlying transformation in Griffith’s experiment was DNA (Avery et al. 1944) or how prokaryotes shuttle DNA from one cell to another. Lederberg and Tatum (1946) discovered conjugational transfer of DNA between *Escherichia coli* cells. Zinder and Lederberg (1952) discovered transduction (phage-mediated gene transfer) during a study of recombination in *Salmonella* strains (Zinder and Lederberg 1952). Gene transfer agents, nonvirulent phage-like DNA-vehicles, were discovered in the early 1970s (Marrs 1974; Solioz et al. 1975). Those mechanisms of gene transfer in prokaryotes turned out to be the mechanisms of prokaryote genetics. The process of generating new gene combinations in prokaryotes is not a process of reciprocal gene exchange, it is a process of unidirectional spread of genes from donors to recipients.

While that was going on, Zuckerkandl and Pauling (1965) proposed that the information stored in DNA, RNA, and proteins could be used for evolutionary research and shortly thereafter, Fitch and Margoliash (1967) showed how one can study evolution using sequences with their method for phylogenetic tree reconstruction from protein sequences. But when protein sequences for cytochrome *c* from many prokaryotic species started to accumulate, it soon became evident that lateral gene transfer (LGT) had probably affected some genes at some point during their history (Ambler et al. 1979; Dickerson 1980; Woese et al. 1980). Genome sequences left no doubt that LGT was a normal component of prokaryote genome evolution. That ushered in an era of discussions among evolutionary biologists about the extent of LGT during microbial evolution (and its implications for the feasibility of a “true” microbial species tree) (Doolittle 1999; Martin 1999; Ochman et al. 2000).

One very popular approach emerged for handling the occurrence of LGT in studies of prokaryote evolution: Remove all the genes from the data that are not universally distributed among genomes or that have obviously been affected by LGT and that hence do not fit the model of a bifurcating phylogenetic tree (e.g., Ciccarelli et al. 2006). Others were more concerned with trying to modify the phylogenetic model to fit the process of genome evolution as it occurs in prokaryotes. That brought networks into the study of microbial genome evolution. Networks do not impose a tree structure on the data from prokaryote genomes. Networks allow evolutionary biologists to study the history of all the genes in prokaryote genomes, not just the ones that fit the model of a bifurcating tree. This brings back into the fold those genes that evolve by LGT and enhance the study of how LGT affects prokaryote chromosome and plasmid evolution (e.g., Beiko et al. 2005; Kunin et al. 2005; Dagan et al. 2008; Halary et al. 2010; Alvarez-Ponce et al. 2013).

Our aims for the SMBE (Society for Molecular Biology and Evolution) satellite meeting on reticulated microbial evolution were to get a current summary on the mechanisms for DNA transfer, the importance of LGT in different habitats, and the applicability of networks approaches for the study of microbial genome evolution, flanked by the expectation that a multidisciplinary meeting of scientists working in the fields of molecular evolution, microbial ecology, and networks research will add coherence to the study of reticulated (nontree-like) events during evolution. The meeting took place at the Christian-Albrechts University of Kiel between April 27 and 30, 2014 and included 76 attendants from 17 countries (table 1). The program included 24 invited and 11 contributed oral presentations, in addition to 23 poster presentations.

**Table 1:** Distribution of SMBE Meeting Attendants According to Country of Affiliation and Gender

	Total		Invited Speakers		Contributed Talks		Poster Presentations	
Austria	2		0		1		0	
Canada	1		1		0		0	
Denmark	1		1		0		0	
Finland	3		2		0		1	
France	6		5		2		0	
Germany	39		4		4		15	
Hungary	1		1		0		0	
Ireland	1		1		0		0	
Israel	4		2		1		0	
Italy	1		1		0		0	
Japan	1		0		1		0	
Netherlands	3		1		0		2	
Norway	2		1		0		1	
Sweden	2		0		0		2	
Switzerland	2		1		1		0	
United Kingdom	3		0		1		1	
United States	4		3		0		1	
Total	76		24		11		23	
Male	44	58%	18	75%	7	64%	14	61%
Female	32	42%	6	25%	4	36%	9	39%

Networks themselves were the topic of several presentations focusing mainly on higher-order structure and clustering methods. **Aaron Clauset** (University of Colorado, USA) introduced the inference of large-scale patterns in networks and presented the stochastic block module modularity inference method as applied to *Plasmodium falciparum* gene recombination networks. **Santo Fortunato** (Aalto University, Finland) in his presentation addressed different methods for modularity detection in networks. In his talk, he compared methods employing global versus local optimization of the modularity function and argued in favor of the latter. **Gergely Palla** (Eötvös University, Hungary) presented an algorithm for extracting large-scale hierarchy from tags information (i.e., entity properties) and exemplified its applicability for the analysis of gene ontology terms as well as IMDb and Flickr databases. **Laurent Viennot** (Paris Diderot University, France) presented a network-based algorithm for protein module prediction based on BLAST results and applied it for a study of families of shared genetic fragments across the three domains, plasmidome and virome. **Christian von Mering** (University of Zürich, Switzerland) addressed the challenges of OTU construction from 16S rRNA sequences. A comparative benchmark of various clustering algorithms revealed surprising differences in their results. Exact clustering approaches were recommended.

Several talks focused on the study of LGT mechanisms including transformation, conjugation, and transduction. **Kaare Nielsen** (University of Tromsø, Norway) presented a study of transformation in *Acinetobacter baylyi* revealing frequent uptake and recombination of short DNA sequences (∼5–10 bp). Studies of gene transfer through conjugation are commonly focused on plasmid ecology and evolution. **Kornelia Smalla** (Julius Kühn Institute, Germany) addressed the issue of antibiotic resistance gene spread through mobile genetic elements due to the application of manure for fertilization in agricultural ecosystems. **Eva Top** (University of Idaho, USA) showed phylogenetic evidence for both vertical inheritance and lateral transfer during plasmid evolution. Further data from an experimental evolution study demonstrated that plasmid host adaptation involves improved plasmid persistence and fitness costs. **Aya Brown-Kav** (Volcani Institute, Israel) presented a method for plasmid metagenomics sequencing and assembly and its utility for studying plasmid-mediated LGT in the bovine rumen. Phylogenomic analysis of the plasmid sequences revealed that the majority of LGT occurs between closely related species. Another plasmid metagenomics approach was demonstrated by **Søren Sørensen** (University of Copenhagen, Denmark) for the study of plasmid repertoire in rat gut-microbiota. The analysis of plasmid-coding potential resulted in an unprecedented frequency of unknown genes, revealing that our knowledge of the plasmidome is still lacking. Further studies of plasmid host range using cell sorting techniques revealed the existence of “superhosts”—microbial species having promiscuous plasmid intake dynamics.

Presentations of transduction research topics focused on the study of antiphage defense mechanisms. **Rotem Sorek** (Weizmann Institute of Science, Israel) presented a systematic discovery of toxin–antitoxin (TA) systems that is based on their transfer success (i.e., clonability) and further discussed the role of TA systems as a phage defense mechanism. **Ruth Schmitz-Streit** (Christian-Albrechts University, Kiel) reported the results of an extensive small regulatory RNAs study in *Methanosarcina mazei* and their role in antiphage immune system (CRISPR) function.

The conference program included several studies of recombination that concentrated mostly on the evolutionary dynamics of microbial populations at their natural habitats. **Jukka Corander** (University of Helsinki, Finland) presented a toolbox of alignment-free methods for the study of microbial population genomics and their application for a large-scale analysis of thousands of strains. Results from that study reveal recombination barriers among microbial subpopulations as well as transmission paths of antibiotic resistance genes. **Otto Cordero** (ETH Zürich, Switzerland) focused on understanding microbial gene content diversity through the lens of social and ecological interactions. Ecological populations are not clonal, encoding for diverse gene content. Recombination among strains may lead to transient cooperativity patterns within the community where secretors of beneficial compounds and their consumers may exchange roles. **Jesse Shapiro** (University of Montreal, Canada) presented a study of *Vibrio cholera* evolution within the human host. The results present a surprising level of intrahost variability with most changes occurring within mobile elements. **Yael Artzy-Randrup** (University of Amsterdam, the Netherlands) developed a mathematical model that quantifies the impact of recombination on antigenic variation in *Plasmodium falciparum*. Results from the model suggest that the strain fitness should be measured relative to the fitness landscape of coexisting recombinant strains.

Several speakers examined the impact of LGT on the evolution of microbial populations. **Nobuto Takeuchi** (University of Tokyo, Japan) developed a population genetics model to study the impact of DNA acquisition on the maintenance of genomic information in microbial populations. His results suggest that a sufficient amount of DNA acquisition, even from dead cells, can prevent genomic deterioration over time and rescue prokaryotic populations from Muller’s ratchet. **Rene Niehus** (University of Oxford, England) presented a mathematical model that quantifies genomic diversity in microbial populations. His results demonstrate that even low rates of gene transfer, when coupled with the acquisition of selective traits, may lead to great diversity within the population. **Hande Acar** (IST, Austria) presented a method to calculate the fitness of a single acquired gene within an experimental evolution setup.

The ecology and evolution of host-related microbes has been described from several angles. **Xiangyi Li** (Max Planck Institute for evolutionary biology, Germany) developed a mathematical model to study the interactions among microbial populations. Her results demonstrate the implication of colonization dynamics for the evolution of host-related microbial communities. **Jillian Petersen** (Max Planck Institute for Marine Microbiology, Germany) presented a research of evolution by LGT in the deep-sea *Bathymodiolus* mussels microbial symbionts. Phylogenetic and genomic evidence suggests that the sulfur-oxidizing symbiont has acquired several toxin-related genes, which might be required for the symbiotic relations with the mussel. **Itzhak Mizrahi** (Volcani Institute, Israel) presented a comparative genomics study of methanogenic archaea isolated from the bovine rumen and their free-living sibling species. The symbiotic-specific genes were identified as putative acquisitions from eubacterial donors and their conserved functional domains indicate that they could be important for host-symbiont recognition. **Hinrich Schulenburg** (Christian-Albrechts University of Kiel, Germany) presented an experimental host–parasite evolutionary study of *Caenorhabditis elegans*–*Bacillus turingiensis* coevolution. The study revealed frequent lateral transfers of a toxin-encoding gene, which may contribute to strain diversification and parasitic persistence within the host. **John Baines** (Christian-Albrechts University of Kiel, Germany) presented a study of host–microbe adaptation focusing on the blood-group-related gene *B4galnt2*. An evolutionary study of the *B4galnt2* locus in house mice shows that the gene is under strong purifying selection. A knockout of *B4galnt2* revealed that its expression has an impact on the mouse gut microbiota biodiversity suggesting an important role of *B4galnt2* expression in modulating the symbiotic microbial population.

Phylogenomic studies addressed genome evolution at different levels, from a focus on protein families, mobile elements, whole genomes, and interdomain transfers. **Eugene Koonin** (National Center for Biotechnology Information, USA) presented a comparative gene gain–loss analysis of microbial gene families. The results reveal that gene gain and loss frequencies are proportional to protein divergence and that LGT is a dominant force during microbial evolution. **James McInerney** (National University of Ireland, Ireland) presented the use of sequence similarity networks (SSN) to study protein evolution. His presentation included examples of gene fusions and domain reshuffling which constitute cases of multiple ancestral origins that could not be reconstructed using trees but require a new type of network model—multirooted trees. **Eric Bapteste** (Pierre and Marie Curie University, France) presented the application of SSNs to study introgressive descent (e.g., recombination, endosymbiotic and lateral gene transfer) and highly divergent homologs. Such networks are, for example, useful for the study of composite genes in viral genomes and for the detection of environmental variants of key cellular genes in metagenomic samples. **Tal Dagan** (Christian-Albrechts University Kiel) addressed the application of networks approach to phylogenomic studies of gene transfer and the kinds of barriers to LGT that they reveal. **Marco Fondi** (University of Florence, Italy) presented a study of plasmid evolution using SSNs revealing interesting links of antibiotic resistance genes. SSN reconstructed from metagenomes revealed no correlation between shared genes and geographical distance. **Krister Swenson** (Institute for Computational Biology Montpellier, France) presented a graph-based computational algorithm to reconstruct recombination events during bacteriophage evolution. **Thorsten Klingen** (Heinrich-Heine University of Düsseldorf, Germany) presented a network-based approach for the inference of assortment events during the evolution of influenza A viruses. The method is based on a parsimonious algorithm to join trees reconstructed for specific genomic segments into a network. **Antoine Branca** (Paris-Sud University, France) presented a study of LGT in *Penicillium* species used in the French cheese industry. The genome sequencing of representative species reveals gene blocks that have been putatively acquired by LGT; the distribution of transposable elements can help in several cases to trace the donor species.

Several speakers demonstrated the contribution of interdomain LGTs to genome evolution. **Purificación Garcia-Lopez** (Paris-Sud University, France) addressed the abundance of interdomain gene transfer as observed in metagenomic data sampled from deep-sea habitats. **Bill Martin** (Heinrich-Heine University Düsseldorf, Germany) presented a phylogenomic study on the contribution of eubacterial genes to the evolution of main archaeal groups. **Philippe Lopez** (Pierre and Marie Curie University, France) demonstrated the utility of networks for the study of eukaryotic origins using topological features of SSN constructed for representative genomes from the three domains of life.

Best poster prizes were awarded as follows. First prize: **Shijulal Nelson-Sathi** (Heinrich-Heine University Düsseldorf, Germany) who presented a poster on archaeal genome evolution. Second prize: **Manu Tamminen** (University of Helsinki, Finland) whose poster demonstrated the application of single cell metagenomics. Third prize: **Bram van Dijk** (Utrecht University, the Netherlands) who presented a poster entitled “Evolution of differential rates of horizontal gene transfer.”

In summary, the speakers at the meeting presented a wide range of studies focusing on different aspects of LGT within the prokaryotic domain. Importantly, contemporary LGT research is conducted at multiple levels, from the very basic molecular mechanisms that are involved in the acquisition—or defense against—foreign DNA, to large-scale high-resolution studies of descent with recombination in natural microbial populations residing in various habitats. Further theoretic studies are bridging the gap between existing models for population genetics and the consequences of LGT during microbial evolution. The emerging picture from the conference revealed that the kinds of information supplied by current genome sequencing methods have surpassed the existing theory and methods for evolutionary reconstruction from molecular data. Major challenges in the field include the ability to reconstruct reticulated events during the evolution of genes, genomes, and populations, and the scalability of methods to data sets of ever increasing size and complexity. Promising research directions include the application of short sequences (k-mer)-based approaches for phylogenetic reconstruction and the transition from bifurcating trees toward the networks framework.
